# Fermionic Free Energies from Ab Initio Path Integral Monte Carlo Simulations of Fictitious Identical Particles

**DOI:** 10.1021/acs.jctc.5c00301

**Published:** 2025-07-24

**Authors:** Tobias Dornheim, Zhandos Moldabekov, Sebastian Schwalbe, Panagiotis Tolias, Jan Vorberger

**Affiliations:** † Center for Advanced Systems Understanding (CASUS), D-02826 Görlitz, Germany; ‡ 28414 Helmholtz-Zentrum Dresden-Rossendorf (HZDR), D-01328 Dresden, Germany; § 156318 Royal Institute of Technology (KTH) Stockholm, SE-100 44 Stockholm, Sweden

## Abstract

We combine the recent η-ensemble path integral Monte Carlo approach to the free energy [Dornheim et al. *Phys. Rev. B* **2025** *111*, L041114] with a recent fictitious partition function technique based on inserting a continuous variable that interpolates between the bosonic and Fermionic limits [Xiong and Xiong *J. Chem. Phys.* **2022** *157*, 094112] to deal with the Fermion sign problem. As a practical example, we apply our setup to the warm, dense, uniform electron gas over a broad range of densities and temperatures. We obtain accurate results for the exchange–correlation free energy down to half the Fermi temperature and find excellent agreement with the state-of-the-art parametrization by Groth et al. [*Phys. Rev. Lett.* **2017** *119*, 135001]. Our work opens up new avenues for the future study of a host of interacting Fermi systems, including warm dense matter, ultracold atoms, and electrons in quantum dots, and for Fermionic free energy calculations with unprecedented system size.

## Graphical abstract

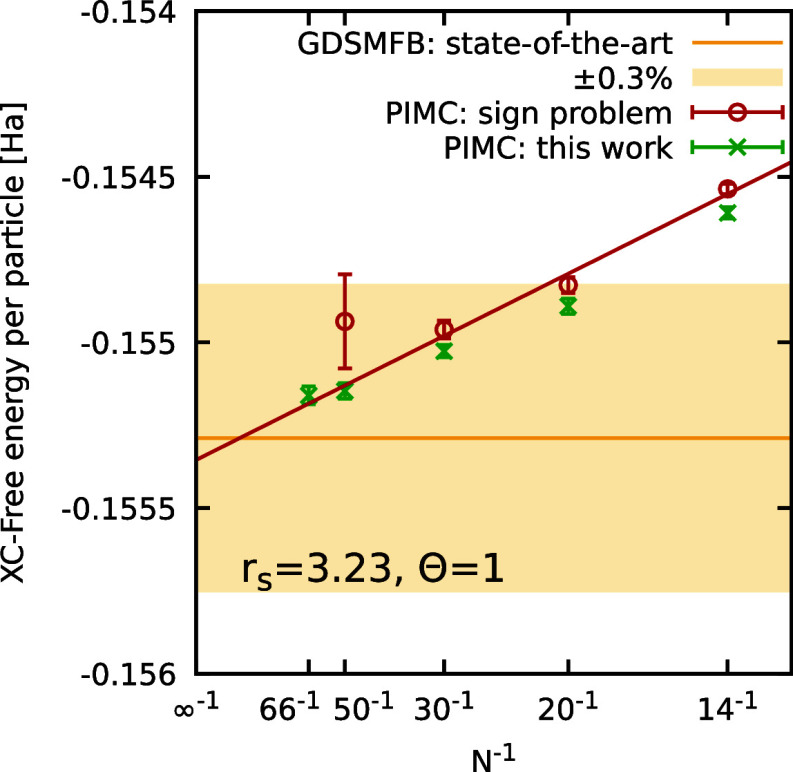

## 1 Introduction

The ab initio path integral Monte Carlo (PIMC) technique^1^ constitutes one of the most important methods for the simulation of interacting quantum many-body systems within physics, quantum chemistry and related disciplines. Based on Feynman’s imaginary-time path-integral formalism,^2^ modern sampling schemes facilitate quasi-exact (asymptotically exact within the given Monte Carlo error bounds) simulations of *N* ∼ 10^4^ bosons,^3,4^ which has substantially advanced our understanding of fundamental collective effects such as superfluidity and Bose–Einstein condensation.^1,5,6^ In practice, PIMC simulations give one direct access to a host of physical observables, including pair correlation functions and static structure factors,^1,7,8^ different energy contributions and pressures,^7,9,10^ as well as linear and nonlinear static density response properties.^11–14^ Moreover, the analytic continuation of various imaginary-time correlation functions allows, in principle, for the estimation of dynamic properties,^15–20^ although the required inversion, e.g., of a two-sided Laplace transform, constitutes a notoriously difficult inverse problem.^21^ While the direct estimation of the free energy *F* = – β^–1^log­(*Z*), where *Z* = *Z*(*N*, Ω, β) and β = 1/*k*_B_ *T* are the canonical partition function (assuming a cubical simulation cell of volume Ω = *L* ^3^) and inverse temperature, is not possible, the introduction of generalized configuration spaces^3,22–24^ allows for the straightforward estimation of free energy differences, e.g., between the interacting system of interest and a noninteracting reference system for which the free energy is already known exactly.^25^

Unfortunately, PIMC simulations of quantum degenerate Fermions are afflicted with the *Fermion sign problem* (FSP);^26–28^ it leads to an exponential increase in the required compute time e.g., with increasing *N* or decreasing *T*. Thus, the FSP often prevents the direct utilization of PIMC to study the wealth of interesting physical phenomena in interacting many-Fermion systems, such as the formation of Wigner molecules^29,30^ and Wigner crystals at low densities^31,32^ and the exchange–correlation (XC) induced incipient roton feature in strongly coupled Fermi liquids at intermediate wavenumbers.^18,33–36^ Due to the pressing need to accurately describe quantum degenerate Fermions, a number of methodological advances^7,37–45^ have been presented over the past decade that exhibit different strengths and weaknesses; see, e.g., ref 10. for a topical overview.

Here we focus on and combine two interesting recent ideas. The first idea by Xiong and Xiong concerns the circumvention of the FSP in path integral molecular dynamics simulations of Fermions based on the controlled extrapolation over a continuous partition function variable ξ.^46–48^ This idea was subsequently adapted to Fermionic PIMC simulations^49^ and successfully applied to various systems such as the uniform electron gas (UEG),^49,50^ warm dense hydrogen and beryllium,^51–53^ as well as ultracold ^3^He.^54^ On the one hand, this ξ-extrapolation method allows for large Fermionic PIMC simulations of weakly to moderately degenerate Fermions without the exponential scaling with respect to the system size, culminating in simulations of up to *N* = 1000 electrons.^50^ On the other hand, the application of the ξ-extrapolation method to strongly degenerate phase diagram regions has proven to be less straightforward.^47,49^ The second idea by the present authors concerns the utilization of the extended η-ensemble scheme that allows for the estimation of the free energy of quantum many-Fermion systems by combining a number of bosonic, sign-problem free PIMC simulations with the correct Fermionic expectation value of *F* via the *average sign S*.^23,24^ Here, the main limitation is the sufficiently accurate resolution of the average sign *S* that is known to decrease, in leading order, exponentially with decreasing temperature or increasing particle number.^27^

In the present work, we show that it is possible to accurately estimate the average sign *S*, and, consequently, the corresponding difference between the Fermi–Dirac and Bose–Einstein systems of interest, in the Fermionic limit of ξ = −1 based on PIMC simulations for |ξ| < 1 for which the sign problem is substantially less severe. As a result, we are able to obtain accurate results for the XC free energy of the warm dense uniform electron gas (UEG)^55–58^ down to half the Fermi temperature, which was not possible before with coordinate space implementations without using approximate nodal restrictions.^7^ Moreover, our approach facilitates the simulations of comparably large numbers of electrons with very high accuracy around the Fermi temperature. We find excellent agreement with the parametrization by Groth et al.,^56^ which further substantiates the high quality of current UEG representations. Our work opens up new avenues for the future investigation of a gamut of interacting Fermi–Dirac systems, including warm dense matter,^59^ ultracold atoms,^54,60,61^ and electrons in quantum dots.^29,39,46^

The paper is organized as follows: In Section 2, we introduce the relevant theoretical background, including the basic idea behind the PIMC method,^1^ the ξ-extrapolation technique,^46,49^ and the η–ensemble PIMC approach to the free energy.^23,24^ Section 3 contains our new simulation results, starting with an investigation of the ideal Fermi gas (Section 3.1), proceeding with the UEG (Section 3.2) and culminating in new estimates for the XC free energy *F*_XC_ (Section 3.3). The paper is concluded by a summary and outlook in Section 4.

## 2 Theory

### 2.1 Path Integral Monte Carlo

The ab initio PIMC method is based on the celebrated quantum-to-classical isomorphism^62^ that allows one to map the nonideal quantum many-body system of interest onto an effectively classical system of interacting ring polymers. Accessible introductions to PIMC have been presented elsewhere;^1,3,63^ here we restrict ourselves to a discussion of the most important relations. As a starting point, we consider the canonical partition function

$$Z(N,\Omega ,\beta )=\sum_{\sigma }\int dX\xi^{p_{l}(\sigma )}W(X)$$ 1

where **X** is a so-called path configuration that contains the coordinates of all *N* particles on *P* discrete imaginary-time slices and *W*(**X**) is the corresponding configuration weight, an analytically known function that is easy to evaluate in practice. In addition, we have to evaluate the sum over all permutations of particle coordinates σ with ξ = 1 and ξ = −1 corresponding to Bose–Einstein or Fermi–Dirac statistics.^63^ Evidently, when ξ = −1, contributions to eq 1 can be negative for an odd number of pair exchanges *p*_l_(σ), precluding the interpretation of *P*(**X**) = ξ^*p*_l_(σ)^ *W*(**X**)/*Z*(*N*, Ω, β) as a proper probability distribution for Fermions.

As a practical workaround, it is common practice to perform a bosonic PIMC simulation enabled by setting ξ = 1 in eq 1 and subsequently to extract Fermionic expectation values of any observable *Â* as^27^

$$\langle \hat{A}\rangle_{\xi }=\frac{\langle \hat{A}\hat{S}\rangle_{\left|\xi \right|}}{\langle \hat{S}\rangle_{\left|\xi \right|}}$$ 2

The denominator in eq 2 is known as the *average sign S* and constitutes a straightforward measure for the amount of cancellation between negative and positive contributions to eq 1. In fact, it directly corresponds to the ratio of Fermionic and bosonic partition functions,

$$S(\xi )=\frac{Z_{\xi }(N,\Omega ,\beta )}{Z_{\left|\xi \right|}(N,\Omega ,\beta )}$$ 3

In practice, a vanishing sign indicates a vanishing signal-to-noise ratio of eq 2, which is known as the *Fermion sign problem* in the literature.^26,27^ It constitutes an exponential computational bottleneck with respect to important system parameters such as the number of particles *N* or the inverse temperature β, restricting the application of Fermionic PIMC simulations to relatively moderate temperatures.^10^

### 2.2 Simulation of Fictitious Identical Particles

A common workaround to this bottleneck is given by the restricted PIMC method,^61,64^ which, in principle, allows one to avoid the sign problem by imposing restrictions on the nodal structure of the thermal density matrix. While being formally exact, the exact nodal structure for an interacting quantum many-body system is a priori unknown; it is thus approximated in practice e.g. using the nodes of an ideal Fermi gas^7^ or more sophisticated Hartree–Fock nodes.^37^ While the restricted PIMC method has been applied successfully by Militzer and co-workers to a number of warm dense matter systems, see ref 9. and references therein, the accuracy of the thus computed results has fundamentally remained unclear in many cases beyond the UEG.^38,40,41^

Very recently, Xiong and Xiong^46^ have proposed to circumvent the sign problem by performing path integral molecular dynamics simulations of fictitious identical particles with continuous values of the ξ-variable −1 ≤ ξ ≤ 1, cf. eq 1 above. In particular, the simulations are FSP-free for ξ ≥ 0, and the expectation value of any observable should be a smooth function of ξ. The basic idea of the ξ-extrapolation method is then to perform a polynomial fit of ⟨*Â*⟩_ξ_ to extrapolate to the true Fermionic limit of ξ = −1. Subsequently, this idea has been adapted to Fermionic PIMC simulations,^49^ where it has been demonstrated to be capable of giving highly accurate and essentially unbiased results for large systems at weak to moderate degrees of quantum degeneracy.^50^ In particular, it removes the exponential scaling with respect to the number of simulated Fermions *N*, which is very important in its own right. Consequently, the ξ-extrapolation method has been used to compute a gamut of observables including energies, structural properties and even imaginary-time correlation functions, with its applications ranging from electrons in quantum dots to warm dense quantum plasmas and ultracold atoms.^47,48,51–54^

### 2.3 Free Energies from the η-Ensemble

In the present work, we focus on the free energy

$$F(N,\Omega ,\beta )=-\frac{1}{\beta }\log(Z(N,\Omega ,\beta ))$$ 4

which, being equivalent to the partition function itself, is not a straightforward observable for PIMC calculations. Instead, PIMC is generally limited to the estimation of free energy differences between two Hamiltonians *Ĥ*_1_ and *Ĥ*_2_.^22^ A common approach is to relate the interacting system of interest with a noninteracting reference system at the same conditions for which the free energy can be computed semianalytically in absolute terms.^25^ This, however, is tremendously impractical in the case of Fermions as PIMC simulations of ideal Fermi systems are afflicted with a stifling sign problem.^27^ As a practical workaround, Dornheim et al.^23,24^ have recently suggested a different route where the free energy of the interacting Bose system is first evaluated with respect to the ideal Bose gas and then the free energy of the interacting Fermi system of interest is recovered from a Fermionic PIMC simulation, where the FSP is substantially less severe. In this approach, the free energy of the interacting Fermi system is given by^23^

$$F_{F}=F_{B,0}-\frac{1}{\beta }\left\{\overset{N_{\eta }}{\underset{i=1}{\sum }}\log\left(r\left(\eta_{i-1},\eta_{i}\right)\right)+\log\left(S\right)\right\}$$ 5

where *F*_B,0_ denotes the free energy of a noninteracting Bose system at the same conditions, the first sum in the curly brackets connects the noninteracting (i.e., η = 0) with the interacting (i.e., η = 1) limits in the bosonic sector with *r*(η_*i*–1_, η_*i*_) being the ratio of partition functions for adjacent η-values (with ··· < η_*i*–1_ < η_*i*_ < η_*i*+1_ < ···) and the second term in the curly brackets relates the free energies of interacting Fermions and bosons;^23,24^ the corresponding generalized Hamiltonian is given by

$${\hat{H}}_{\eta }=\hat{K}+\eta \hat{V}$$ 6

where *K̂* and *V* ^ denote the kinetic and potential contributions to the full Hamiltonian *Ĥ*, which is recovered in the limit of η = 1. From the perspective of the Fermion sign problem, the only problematic contribution in eq 5 is given by the sign *S* = *S*(−1) [cf. eq 3], which vanishes within the given Monte Carlo error bars for too large systems or too low temperatures.

For completeness, we note that eq 5 is generally considerably more convenient than usual thermodynamic integration (i.e., adiabatic connection^65,66^ ) routes to the free energy. Specifically, we work directly with free energies and, in principle, only require a single simulation connecting the interacting and ideal limits without the need for intermediate state points required for the integration, e.g., over a continuous coupling constant η.

### 2.4 Free Energies from Fictitious Identical Particles

The aim of the present work is to generalize the spirit behind the ξ-extrapolation method to the computation of Fermionic free energies via eq 5. To this end, and without loss of generality, we make a simple exponential ansatz for *S*(ξ),

$$S(\xi \leq 0)=e^{a(\xi \leq 0)\xi }\Rightarrow a(\xi \leq 0)=\frac{\log[S(\xi \leq 0)]}{\xi }$$ 7

This is motivated by the recent observation that the sign exponentially decays with |ξ| at some parameters,^49^ in which case the function *a*(ξ ≤ 0) will be a simple constant. The latter case would indicate a linear crossover in the free energies of the bosonic and Fermionic limits with respect to ξ, see also eq 10 below. Crucially, any known functional dependence of *a* on ξ would allow us to extrapolate to the true Fermionic limit of the sign *S*(−1) from PIMC results for *S*(ξ < 0) at smaller |ξ|, for which the sign can be resolved with feasible computational effort.

In practice, we propose an empirical expansion of the form

$$a(\xi )=c_{0}+c_{1}\xi^{3/2}+c_{2}\xi^{1/2}$$ 8

which works well for the conditions explored in the present work.

## 3 Results

All PIMC results that are presented in this work have been obtained using the open-source ISHTAR code,^67^ and are freely available online.^68^ We use *P* = 200 primitive imaginary-time propagators throughout, and the convergence with *P* has been carefully checked. Note that we assume Hartree atomic units.

### 3.1 Ideal Fermi Gas

We start our investigation with the ideal Fermi gas, which, as we shall see below, exhibits the same qualitative trend as the interacting UEG. In particular, in Figure 1, we show *a*(ξ)/*N* as a function of the system size *N* for ξ = −1 and ξ = −0.2 at Θ = 1 (left) and Θ = 0.5 (right). We note that, for the ideal system at constant Θ, neither the average sign *S*(ξ < 0) nor the sign exponential scaling factor *a*(ξ < 0) depend on the density. The red solid curves correspond to the exact Fermionic limit of *a*(−1)/*N*, which have been obtained by combining the noninteracting limit of eq 3 with semianalytical results for the partition function of the ideal Bose gas and the ideal Fermi gas.^24,25^ We observe a monotonic and nonmonotonic dependence on *N* for Θ = 1 and Θ = 0.5, respectively, which is an immediate consequence of the corresponding finite-size effects in the noninteracting free energy. In fact, it is easy to see that the value *a*(−1) directly relates to the free energy difference between the bosonic and Fermionic limits,

$$F_{F}-F_{B}=\frac{a(-1)}{\beta }$$ 9

The green crosses show PIMC results for *a*(−0.2)/*N*. For the moderately quantum degenerate case of Θ = 1, we find excellent agreement between *a*(−0.2) and *a*(−1), which substantiates the previous observation that *a*(ξ < 0) = *a* = *const* at these conditions. In the spirit of eq 9, the combination of eqs 2and[Disp-formula eq5]) with eq 7 implies that, for any state point, the free energy difference between its Fermionic sector ξ < 0 image and its bosonic sector −ξ > 0 image is a linear function of the ξ variable.

$$F(\xi <0)-F(-\xi >0)=-\frac{a(\xi <0)}{\beta }\xi ≃-\frac{a}{\beta }\xi$$ 10

In practice, this gives us an exponential acceleration of our PIMC simulations as we only have to resolve *S*(ξ) for small absolute values of ξ in order to estimate its true Fermionic limit of *S*(−1) with very high precision. For Θ = 0.5, on the other hand, we observe significant deviations between *a*(−0.2) and *a*(−1), which are particularly pronounced for small *N*. A possible explanation for this trend is based on the related length scales: the physical behavior of the system can be expected to intrinsically change with the variable ξ when the box length *L* is comparable to the thermal wavelength $\lambda_{\beta }=\sqrt{2\pi \beta }$ , which would explain the seeming convergence of *a*(−0.2) toward *a*(−1) at large *N*.

**Figure 1:**
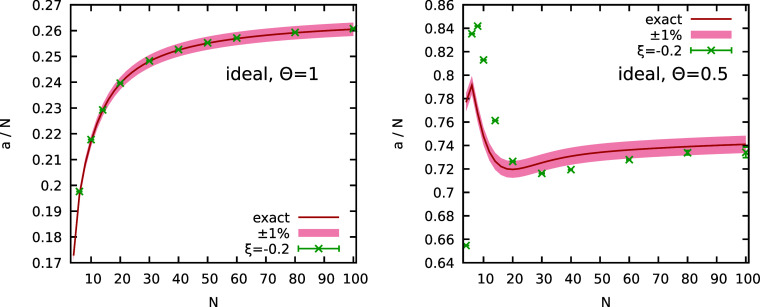
Dependence of the sign exponential scaling factor per particle *a*/*N* of the unpolarized ideal Fermi gas on the number of particles *N* at Θ = 1 (left) and Θ = 0.5 (right). The red lines depict the exact semianalytical results for the true Fermionic limit *a*(ξ = −1) computed from the noninteracting Fermionic average sign *S*_0_,^24^ while the green crosses correspond to PIMC results for *a*(ξ = −0.2) at the same conditions. In both panels, the shaded red area indicates an interval of ±1% around the exact Fermionic limit that has been included as a guide-to-the-eye.

### 3.2 Uniform Electron Gas

In Figure 2, we investigate the sign exponential scaling factor *a*(ξ) of the warm dense UEG with *N* = 8 at *r*_s_ = 3.23. Such a moderate coupling can be realized in experiments with hydrogen jets.^69–71^ The blue diamonds and crosses show results for the UEG and the ideal Fermi gas, respectively, at Θ = 1, i.e., for a moderate degree of quantum degeneracy. We find a negligible dependence of *a*(ξ) on ξ at these conditions for both systems, consistent with the findings from Figure 1; see also the right panel of Figure 2 for a magnified segment around the UEG results at Θ = 1 (bottom points). The higher *a*(ξ) values for the ideal Fermi gas compared to the interacting UEG are a consequence of the increased degeneracy and, consequently larger free energy difference or equivalently the smaller average sign in the former case. The green data points show a similar investigation at Θ = 0.75. In this case, we find a very small dependence of *a*(ξ) on ξ (of the order of ∼1% for small ξ), which can be fitted well by the empirical function

$$a(\xi )=c_{0}+c_{1}|\xi {|}^{3/2}$$ 11

see the solid green curves in Figure 2. In fact, this fit has been constructed on the basis of data only for ξ ≥ −0.4, yet it nicely reproduces the full PIMC data set. In other words, data in this limited ξ-range are sufficient to accurately estimate the true Fermionic limit. For completeness, we have also obtained a data set where we add the corresponding ideal Fermi gas correction to the PIMC results for *a*(ξ) of the UEG

$$\Delta a_{0}(\xi )=a_{0}(-1)-a_{0}(\xi )$$ 12

The results are included as the green squares into Figure 2, but, in this case, they do not constitute an improvement over the raw PIMC results for the UEG; this clearly illustrates the complex interplay of interaction and quantum degeneracy effects that is often cited as a defining feature of the warm dense matter regime.^72^ The red data points show results at Θ = 0.5. In this more strongly degenerate quantum case, we find a more substantial dependence of *a*(ξ) on ξ for both the ideal Fermi gas and the UEG that appear to qualitatively resemble each other. The addition of the eq 12 correction does constitute an improvement for these parameters, but a residual error of ∼1% remains e.g., for ξ = −0.2. On the other hand, the advantage of this approach is that it only requires PIMC results for a single ξ-value as the correct Fermionic limit of the ideal Fermi gas is a priori known; no extrapolation over ξ is needed. The use of eq 11 for ξ ≥ −0.4 to extrapolate to the Fermionic limit of ξ = −1 gives the dashed black curve, which closely, but not exactly matches the correct limit. The addition of the extra term *c*_2_ξ^1/2^ to the fit, cf. eq 8 above, using the same fit interval of ξ ≥ −0.4 gives the solid red curve, which constitutes an even better match. In practice, we advocate that both fits are performed and that the resulting difference is employed as an empirical measure of the extrapolation error.

**Figure 2:**
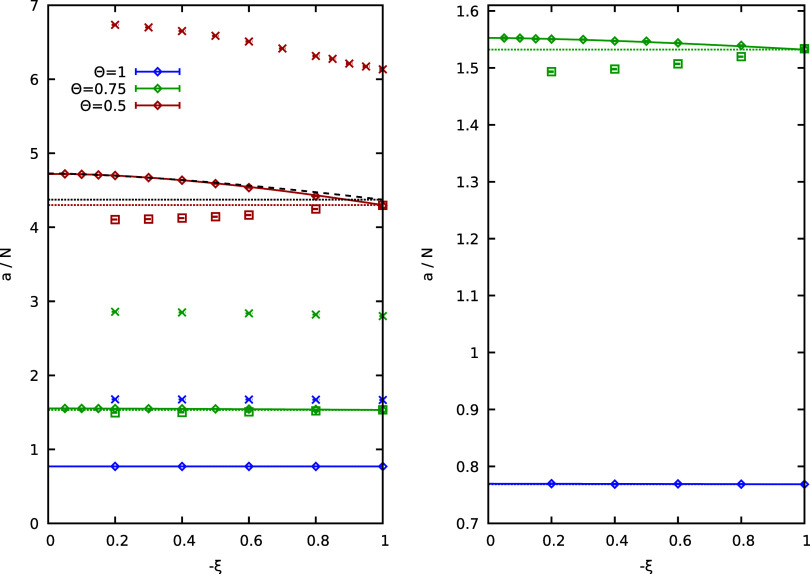
Dependence of the sign exponential scaling factor per particle *a*/*N* of the warm dense paramagnetic UEG on the partition function parameter ξ for *N* = 8 at *r*_s_ = 3.23 and Θ = 1 (blue), Θ = 0.75 (green), Θ = 0.5 (red). The diamonds and crosses have been obtained for the interacting UEG and ideal Fermi gas, respectively, while the squares have been obtained by adding the ideal correction Δ*a*_0_(ξ) to the UEG data, see eq 12. The curves and dotted horizontal lines correspond to different fits that are discussed in the main text. The right panel corresponds to a magnified segment of the left panel.

Next we consider the effect of the density parameter *r*_*s*_ on the behavior of *a*(ξ), which we investigate in the left panel of Figure 3 for the most challenging case of Θ = 0.5 for *N* = 6. The yellow crosses show results for the ideal Fermi gas which correspond to the *r*_s_ → 0 limit of the UEG; the dashed horizontal yellow line is the exact semianalytical result for *a*(−1), which matches the respective PIMC data point, as expected. The blue, green, and red data sets have been obtained from PIMC simulations of the UEG at *r*_s_ = 0.5, *r*_s_ = 3.23, and *r*_s_ = 10. These conditions are respectively realized in laser compression experiments at the National Ignition Facility (NIF),^73,74^ the aforementioned hydrogen jet experiments and the margin of the strongly coupled electron liquid regime.^18,75^ First, we find that *a*(ξ) monotonically decreases with increasing *r*_s_ as the formation of permutation cycles becomes increasingly suppressed by Coulomb repulsion.^63^ Second, we find that *a*(ξ) exhibits an increasingly sharp drop for large |ξ| with a decrease in the degree of nonideality. This indicates an increasing breakdown of the linear expression for the free energy difference between bosonic sector and Fermionic sector images [cf. eq 10] with increasing |ξ|, as the system becomes shaped more profoundly by quantum statistics. Third, we consider the diamonds, which have been obtained by adding the ideal correction, eq 12, onto the raw PIMC results for *a*(ξ). Overall, the correction works reasonably well and removes the bulk of the original dependence on ξ; yet, small residual errors of the order of ∼1% remain, see also the shaded areas indicating an interval of ±1% around the true *a*(−1) Fermionic limit. Finally, the right panel of Figure 3 compares PIMC simulation results for different particle numbers *N* for *r*_s_ = 0.5 and Θ = 0.5. Here, the squares, crosses and diamonds correspond to the ideal Fermi gas, the raw UEG, and the corrected [cf. eq 12] UEG. As a general trend, we find that the ξ-dependence exhibits a less steep drop around large |ξ| with increasing number of particles, which fits to the trends observed in the right panel of Figure 1. In general, the correction works well and we obtain results within an interval of ±1% of the true Fermionic limit (shaded colored areas).

**Figure 3:**
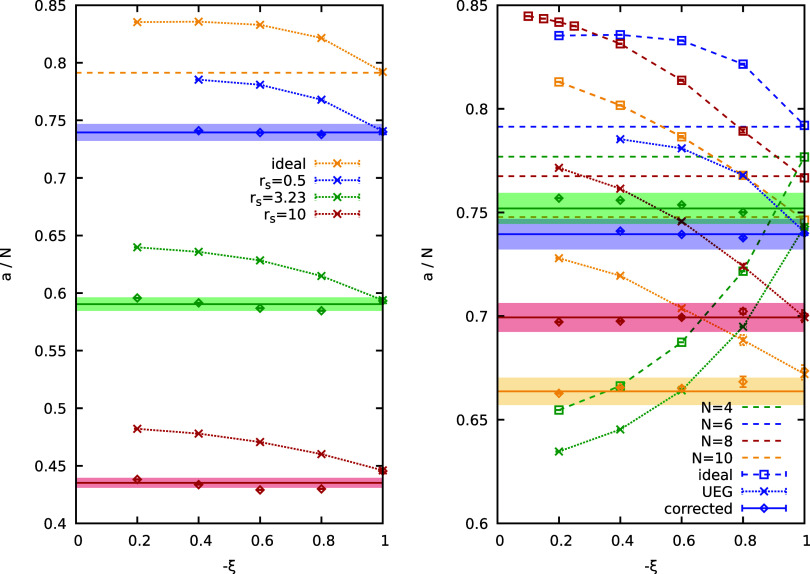
Left panel: the sign exponential scaling factor per particle *a*/*N* of the UEG for *N* = 6 at Θ = 0.5 and *r*_s_ → 0 (yellow), *r*_s_ = 0.5 (blue), *r*_s_ = 3.23 (green), *r*_s_ = 10 (red). The crosses depict raw PIMC results, while the diamonds depict PIMC results obtained by adding the ideal correction Δ*a*_0_(ξ) to the UEG data, see eq 12. Right panel: the sign exponential scaling factor per particle *a*/*N* of the raw UEG (crosses), the ideal Fermi gas (squares) and the corrected UEG (diamonds) at *r*_s_ = 0.5, Θ = 0.5 for different numbers of electrons (*N* = 4, 6, 8, 10). In both panels, the shaded colored areas indicate intervals of ±1% around the exact Fermionic limit of *a*(ξ = −1) (solid lines) that serve as a guide-to-the-eye.

### 3.3 Exchange–Correlation Free Energy

Let us next apply our method to the estimation of the exchange–correlation free energy of the warm dense UEG, *F*_XC_. First and foremost, the advent of accurate, PIMC-based parametrizations of *F*_XC_ ^55–57^ has been of paramount importance for thermal density functional theory (DFT)^76^ simulations of warm dense matter and directly allows for calculations on the level of the local density approximation (LDA).^77–81^ Moreover, they constitute the basis for more sophisticated functionals on higher rungs of Jacob’s ladder of functional approximations.^10,82–84^ In addition, they are important input for a host of other applications^85–87^ and determine both the short- and long-wavelength limit of the exchange–correlation kernel.^66,88–90^

A first important question is given by so-called finite-size effects, i.e., the difference between PIMC simulations for a finite number of particles and the thermodynamic limit that is defined by simultaneously taking the limits of *N* → *∞* and Ω → *∞* with *n* = *N*/Ω being kept constant. Indeed, a vast literature has been dedicated to the study of finite-size effects in the UEG.^8,55,92–95^ An accessible introduction to the finite-size corrections that are being applied in the presented work has been given in ref 55, and we use the efficient and easy-to-use python implementation UEGPY^96^ by F.D. Malone. In the following, all PIMC results have been obtained by subtracting from eq 5 the ideal Fermionic free energy of the *N*-body system *F*_0_(*N*) = −log­[*S*_0_(*N*)]/β and subsequently adding the finite-size correction Δ*F*_XC_(*N*).

In Figure 4, we show our new PIMC results for *F*_XC_/*N* for *r*_s_ = 3.23 and Θ = 0.5 as a function of the partition function parameter ξ for a number of different *N*. The horizontal yellow lines and the surrounding shaded yellow area show the state-of-the-art parametrization by Groth, Dornheim, Sjostrom, Malone, Foulkes, Bonitz (GDSMFB)^56^ and its nominal uncertainty interval of ±0.3% that have been included as a reference. We find the familiar pattern of a significant dependence of *F*_XC_ on ξ, which manifests very differently depending on *N*. The solid red and dashed black curves show the two-parameter [cf. eq 11] and three-parameter [cf. eq 8] empirical fit functions. We find that a reliable extrapolation to the Fermionic limit of ξ = −1 is possible in all cases. For completeness, we note that individual points with larger error bars, such as for *N* = 30, *r*_s_ = 3.23, Θ = 0.5 and −ξ = 0.5 do not meaningfully influence the extrapolation due to their large uncertainty, resulting in negligible weights in the fitting procedure; the large error bars are a direct consequence of the Fermionic cancellation problem. In the top left panel of Figure 5, we show the thus extrapolated results as a function of the inverse system size. The residual finite-size errors are of the order of ≲1%, which is consistent with previous investigations of similar properties.^8,55,95^ Overall, the PIMC results for different *N* are well reproduced by a simple linear fit, which agrees with the given uncertainty range of the GDSMFB parametrization^56^ (yellow area). In the top right and bottom panels, we show finite-size corrected PIMC results for Θ = 0.75 and Θ = 1 at the same density. For these parameters, we find that the full extrapolation procedure shown in Figure 4 is not required, as direct Fermionic PIMC simulations are feasible for many *N*, see the red circles. In addition, the green crosses have been obtained by using *a*(ξ) for ξ = −0.2. The corresponding data have substantially lower error bars due to the less severe FSP. At the same time, we find significant differences for Θ = 0.75, whereas all data are in good agreement for Θ = 1. Generally, we find that the computationally much less demanding calculations with ξ = −0.2 allow for an accuracy of ∼0.1% for Θ = 0.75, which is sufficient for many applications. For Θ = 1, the ξ = −0.2 results are almost indistinguishable from the full calculations with a statistical uncertainty of ∼0.01%, which, to our knowledge, beyond the capabilities of any other reported real-space PIMC implementation so far. Finally, we remark that we find excellent agreement with the GDSMFB parametrization in all cases.^56^

**Figure 4:**
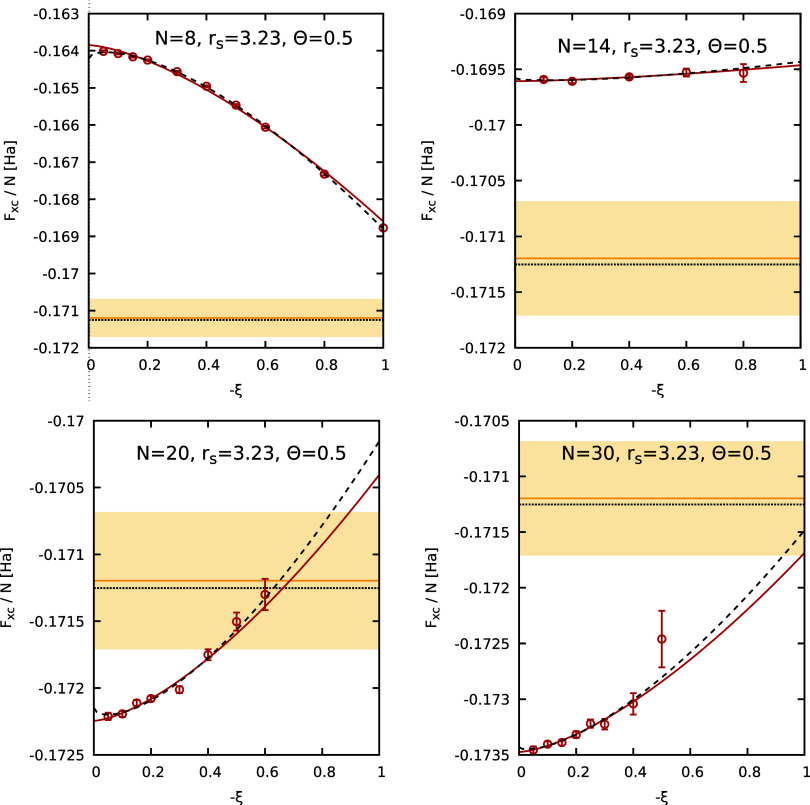
Extrapolation of the XC free energy per particle *F*_XC_/*N* to the Fermionic limit of ξ = −1 for different numbers of particles (*N* = 8, 14, 20, 30) at *r*_s_ = 3.23 and Θ = 0.5. The red circles depict finite-size corrected (FSC) PIMC results, while the solid red and dashed black curves correspond to the two-parameter, see eq 11, and three-parameter, see eq 8, empirical fits. The yellow horizontal line and shaded yellow areas correspond to the GDSMFB parametrization and its respective nominal uncertainty interval of ±0.3%,^56^ and the dotted black line to the UEG parametrization by Ichimaru et al.^91^ (IIT).

**Figure 5:**
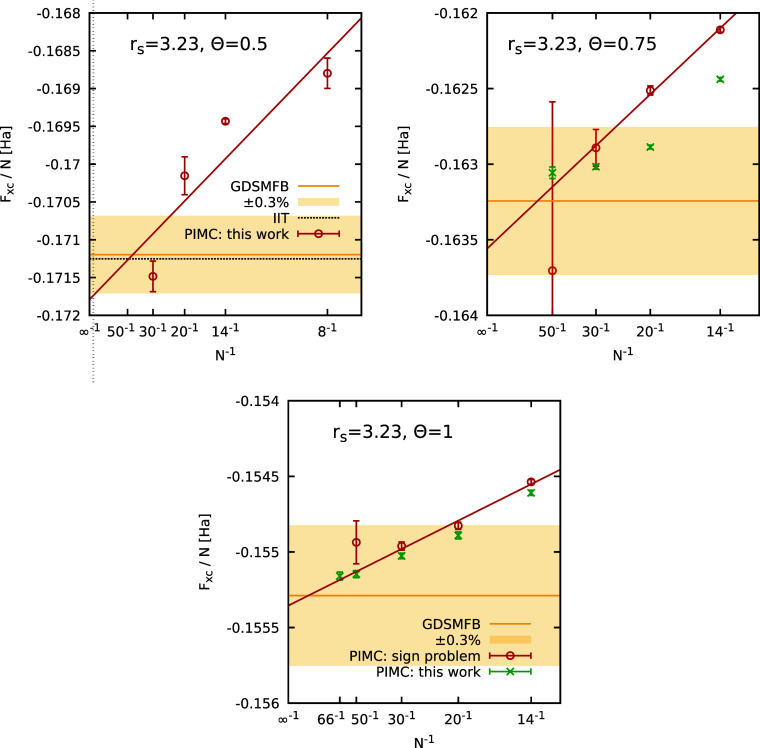
System-size dependence of the PIMC results for *F*_XC_/*N* at *r*_s_ = 3.23 and different temperatures (Θ = 0.5, 0.75, 1). The red symbols in the top left panel (Θ = 0.5) are obtained from an empirical extrapolation to the Fermionic limit (see Figure 4), while the red symbols in the top right (Θ = 0.75) and bottom panels (Θ = 1) correspond to direct PIMC results at the Fermionic limit ξ = −1. In addition, the green crosses correspond to PIMC results using *a*(ξ) with ξ = −0.2. The yellow horizontal line and shaded yellow areas correspond to the GDSMFB parametrization and its respective nominal uncertainty interval of ±0.3%.^56^

Let us next consider the case of *r*_s_ = 10, i.e., the margin of the strongly coupled electron liquid regime. In Figure 6, we show the extrapolation of the finite-size corrected PIMC results for *F*_XC_/*N* to the Fermionic limit of ξ = −1 for different simulated numbers of electrons *N* at the lowest considered temperature of Θ = 0.5. In addition, the blue diamonds have been obtained by applying the ideal correction [eq 12], which, however, does not constitute an improvement in the strongly coupled regime. Overall, we find that a reliable extrapolation to the limit of ξ = −1 is possible in all depicted cases. The corresponding extrapolation to the thermodynamic limit is shown in the top left panel of Figure 8 and is in excellent agreement with the GDSMFB parametrization,^56^ as expected. Moreover, in Figure 7, we investigate the ξ-dependence of our PIMC results for Θ = 0.75, which is negligible. This observation is further substantiated by the top right panel of Figure 8 where the red circles and green crosses show PIMC results obtained using *a*(−1) and *a*(−0.2), respectively. Residual differences between the two data sets are much smaller than residual finite-size effects and can thus be neglected in practice. As before, we find excellent agreement between our new PIMC results for the thermodynamic limit and the GDSMFB parametrization^56^ at both Θ = 0.75 and Θ = 1, as expected.

**Figure 6:**
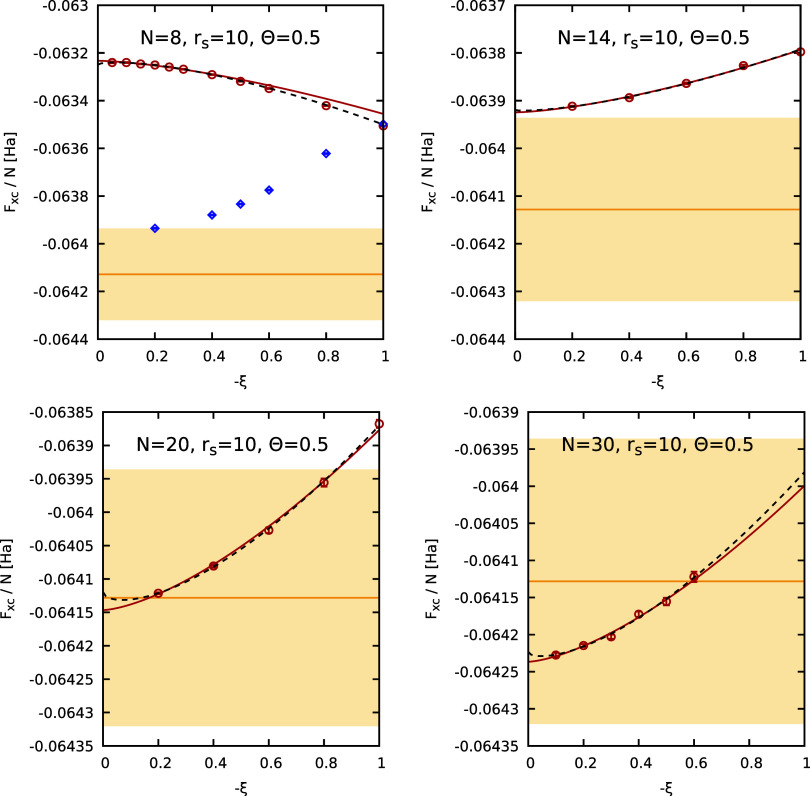
Extrapolation of the XC free energy per particle *F*_XC_/*N* to the Fermionic limit of ξ = −1 for different numbers of particles (*N* = 8, 14, 20, 30) at *r*_s_ = 10 and Θ = 0.5. The red circles depict finite-size corrected PIMC results and the blue diamonds depict PIMC results obtained by adding the ideal correction Δ*a*_0_(ξ) to the UEG data, see eq 12. The solid red and dashed black curves correspond to the two-parameter, see eq 11, and three-parameter, see eq 8, empirical fits. The yellow horizontal line and shaded yellow areas correspond to the GDSMFB parametrization and its respective nominal uncertainty interval of ±0.3%.^56^

**Figure 7:**
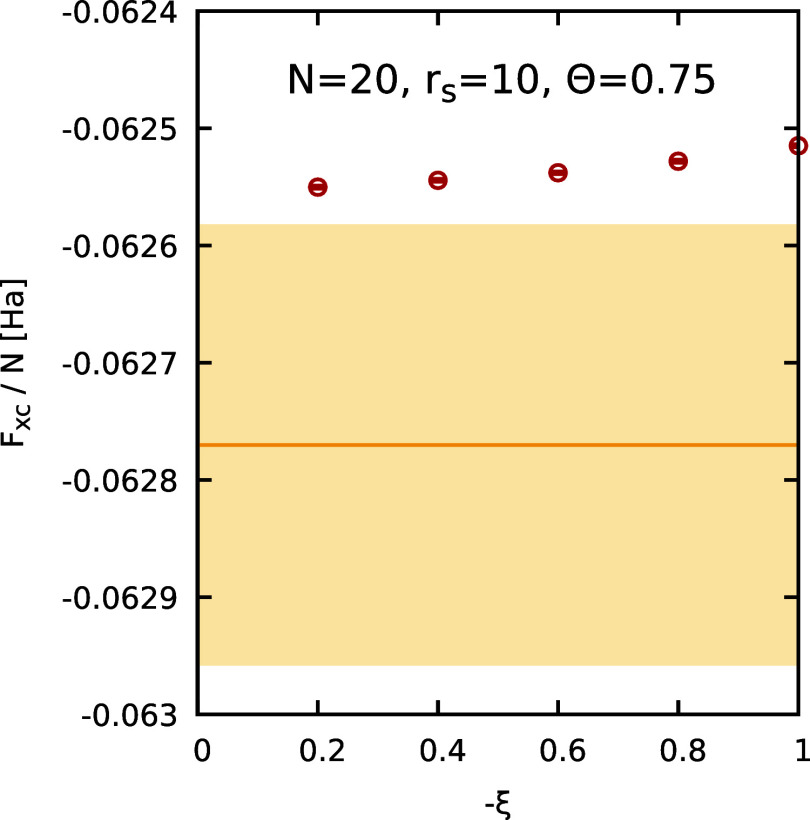
Dependence of the XC free energy per particle *F*_XC_/*N* on the partition function parameter ξ at *r*_s_ = 10, Θ = 0.75, for different particle numbers *N*. The red circles depict finite-size corrected PIMC results. The yellow horizontal line and shaded yellow areas correspond to the GDSMFB parametrization and its respective nominal uncertainty interval of ±0.3%.^56^

**Figure 8:**
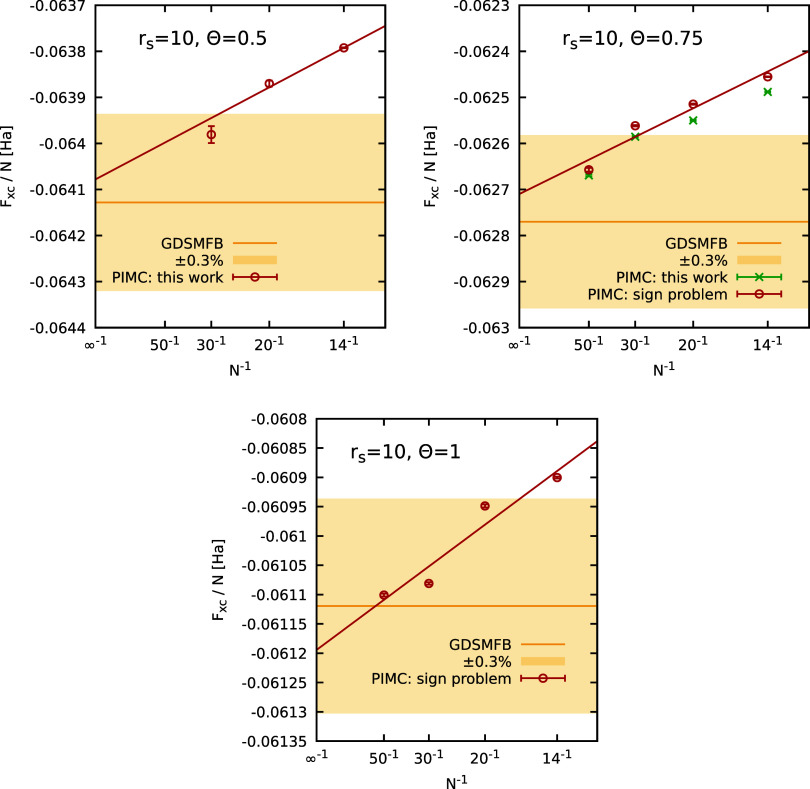
System-size dependence of the PIMC results for *F*_XC_/*N* at *r*_s_ = 10 and different temperatures (Θ = 0.5, 0.75, 1). The red symbols in the top left panel (Θ = 0.5) are obtained from an empirical extrapolation to the Fermionic limit (see Figure 6), while the red symbols in the top right (Θ = 0.75) and bottom panels (Θ = 1) correspond to direct PIMC results at the Fermionic limit ξ = −1. In addition, the green crosses correspond to PIMC results using *a*(ξ) with ξ = −0.2. The yellow horizontal line and shaded yellow areas correspond to the GDSMFB parametrization and its respective nominal uncertainty interval of ±0.3%.^56^

Let us continue our investigation by considering the most difficult high-density regime. In Figure 9, we investigate the ξ-dependence of our finite-size corrected PIMC results at *r*_s_ = 0.5 and Θ = 0.5 for *N* = 8 (left) and *N* = 14 (right). Specifically, the red circles show raw PIMC results for different ξ and the blue diamonds have been obtained by adding the ideal correction (eq 12). Evidently, the correction removes some of the ξ-dependence, but the attained accuracy is not sufficient to meaningfully constrain existing models for *F*_XC_/*N*, cf. the horizontal yellow line corresponding to the GDSMFB parametrization.^56^ Furthermore, we have performed two-parameter [cf. eq 11] and three-parameter [cf. eq 8] empirical fits within the range of −0.4 ≤ ξ < 0 and the results are included as the solid red and dashed black curves, respectively. The latter fit performs particularly well and nicely reproduces our larger ξ PIMC results that were not included into the fit input for *N* = 8. In summary, our new approach is thus indeed capable of giving reasonable results for the free energy in the high-density low-temperature regime, but the accuracy is not high enough for the quantification of the comparably small XC-contribution to the full free energy. In Figure 10, we perform a similar analysis at *r*_s_ = 0.5 and Θ = 0.75. Overall, we find that (i) the ideal correction (eq 12) reduces the dependence on ξ but is not decisive, (ii) the extrapolation to the Fermionic limit works well. The sole exception to the latter observation is given by the dashed black curve that corresponds to the three-parameter empirical fit [cf. eq 8] for *N* = 30, which is evidently spurious. The corresponding extrapolation of the residual finite-size errors to the thermodynamic limit is shown in the left panel of Figure 11, where we again find good agreement with the result of the GDSMFB parametrization.^56^ Finally, in Figure 12, we consider the dependence of our PIMC results for *F*_XC_/*N* on ξ for Θ = 1. At these conditions, the proposed linear expression for the free energy difference between boson and Fermions (eq 10) is very accurate and our results for *a*(ξ) are independent of ξ. Corresponding results utilizing *a*(−0.2) for different *N* are shown in the right panel of Figure 11 and lead to the expected thermodynamic limit.

**Figure 9:**
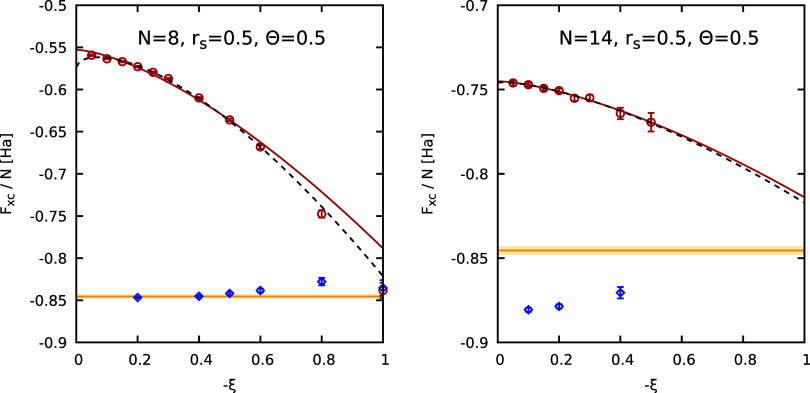
Extrapolation of the XC free energy per particle *F*_XC_/*N* to the Fermionic limit of ξ = −1 for different numbers of particles (*N* = 8, 14) at *r*_s_ = 0.5 and Θ = 0.5. The red circles depict raw PIMC results and the blue diamonds depict PIMC results obtained by adding the ideal correction Δ*a*_0_(ξ) to the UEG data, see eq 12. The solid red and dashed black curves correspond to the two-parameter, see eq 11, and three-parameter, see eq 8, empirical fits. The yellow horizontal line and shaded yellow areas correspond to the GDSMFB parametrization and its respective nominal uncertainty interval of ±0.3%.^56^

**Figure 10:**
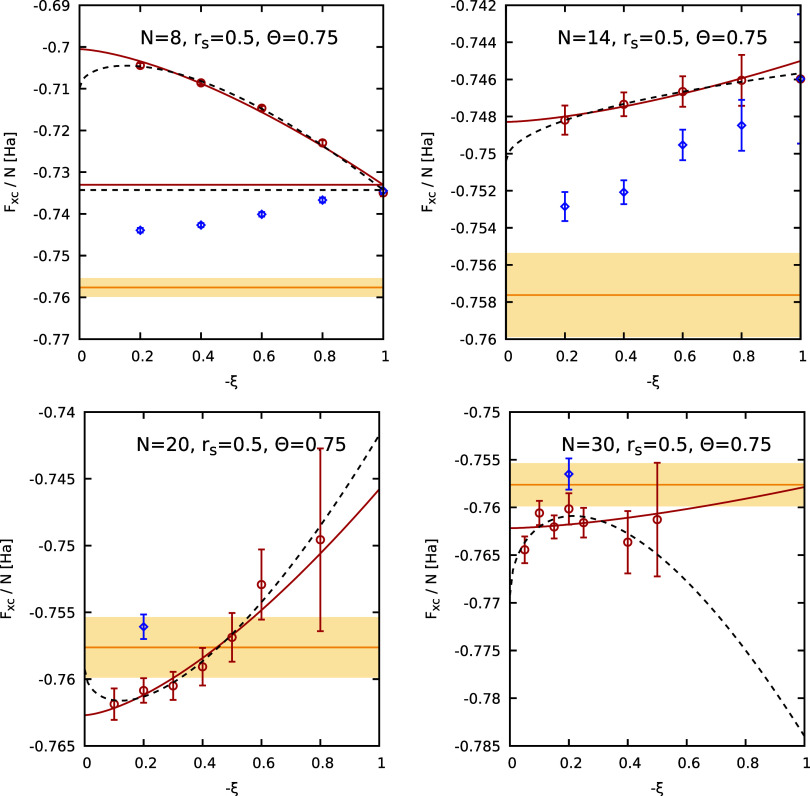
Extrapolation of the XC free energy per particle *F*_XC_/*N* to the Fermionic limit of ξ = −1 for different numbers of particles (*N* = 8, 14, 20, 30) at *r*_s_ = 0.5 and Θ = 0.75. The red circles depict raw PIMC results and the blue diamonds depict PIMC results obtained by adding the ideal correction Δ*a*_0_(ξ) to the UEG data, see eq 12. The solid red and dashed black curves correspond to the two-parameter, see eq 11, and three-parameter, see eq 8, empirical fits. The yellow horizontal line and shaded yellow areas correspond to the GDSMFB parametrization and its respective nominal uncertainty interval of ±0.3%.^56^

**Figure 11:**
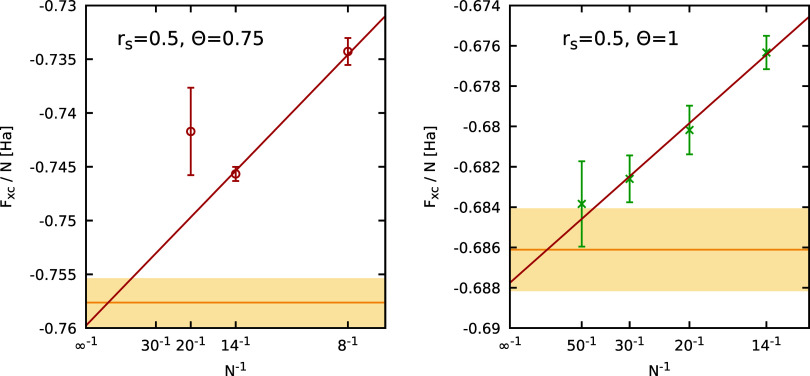
System-size dependence of the PIMC results for *F*_XC_/*N* at *r*_s_ = 0.5 and different temperatures (Θ = 0.75, 1). The red symbols in the left panel (Θ = 0.5) are obtained from an empirical extrapolation to the Fermionic limit (see Figure 10), while the green crosses in the right panel correspond to PIMC results using *a*(ξ) with ξ = −0.2. The yellow horizontal line and shaded yellow areas correspond to the GDSMFB parametrization and its respective nominal uncertainty interval of ±0.3%.^56^

**Figure 12:**
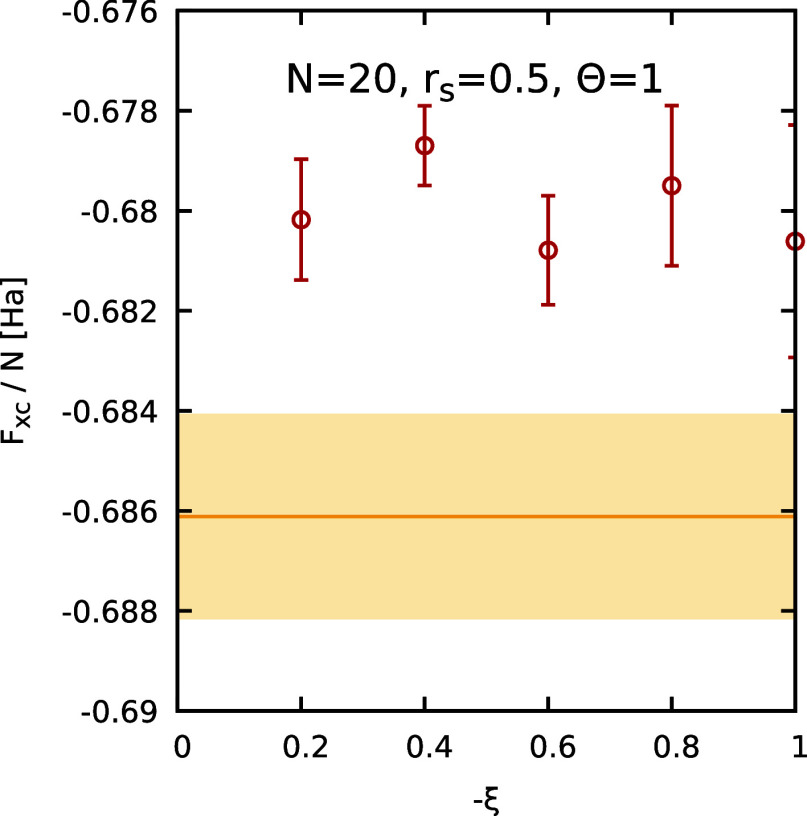
Dependence of the XC free energy per particle *F*_XC_/*N* on the partition function parameter ξ at *r*_s_ = 0.5, Θ = 1, for *N* = 20. The red circles depict finite-size corrected PIMC results. The yellow horizontal line and shaded yellow areas correspond to the GDSMFB parametrization and its respective nominal uncertainty interval of ±0.3%.^56^

## 4 Discussion

We have combined the recent η–ensemble PIMC approach to the free energy^23,24^ with the ξ–extrapolation technique^46,49^ to deal with the Fermion sign problem. This has allowed us to directly compute the XC free energy of the UEG over a broad range of densities and temperatures, leading to the following conclusions. For moderate levels of quantum degeneracy (Θ ≳ 1), the average sign exhibits a simple exponential decay with ξ, leading to a straightforward linear expression for the corresponding free-energy contribution. As a consequence, it is possible to obtain highly accurate results for the free energy in the Fermionic limit (∼0.01%) even for comparably large numbers of electrons with substantially reduced computational cost. For higher levels of quantum degeneracy, i.e., Θ ≲ 0.75, the linearity breaks down. Nevertheless, we find that an empirical extrapolation over ξ works well in most cases. As a consequence, we have been able to obtain accurate results for *F*_XC_/*N* down to Θ = 0.5, which was not possible with previous coordinate space PIMC methods without using approximate nodal restrictions. At the same time, we note that the application of the present methodology to even lower temperatures will not be possible directly due to the Fermion sign problem. Indeed, even at Θ = 0.5, meaningful results were limited to moderate and lower densities.

In addition to being interesting in their own right, our results constitute an independent cross check of available parametrizations of the warm dense UEG in a regime where input data had been particularly sparse.^55,58^ The excellent agreement with the GDSMFB parametrization^56^ thus further substantiates the high quality of our state-of-the-art description of this archetypal model system. Future efforts might include the estimation of the free energy of real WDM systems starting with hydrogen^10,51,97^ and other light elements potentially up to beryllium,^51,52^ where the ξ-extrapolation method apparently performs comparably to the UEG. In addition with a suitable extrapolation to the thermodynamic limit,^94,95,98^ such results might be used to test existing equation of state data tables,^9^ and to compare with experimental measurements, e.g., at the National Ignition Facility^73,74,99^ and the OMEGA laser facility;^100,101^ see also the recent warm dense matter roadmap by Vorberger et al.^72^ and references therein.

Finally, we note that both ingredients to the present method—the η–ensemble approach and the ξ-extrapolation technique—can be applied to any Fermi system, including ultracold ^3^He^54,60,61^ and electrons in quantum dots.^29,39,46,49^ From a methodological perspective, we mention possible future efficiency gains by utilizing clever treatments of the long-range Coulomb interaction^102^ or implemented path contraction schemes^103^ that are common practice in path-integral molecular dynamics simulations.

## Data Availability

The data supporting the findings of this study will be made available on the Rossendorf Data Repository (RODARE) upon publication.
